# Deubiquitinase OTUD5 promotes hepatitis B virus replication by removing K48-linked ubiquitination of HBV core/precore and upregulates HNF4ɑ expressions by inhibiting the ERK1/2/mitogen-activated protein kinase pathway

**DOI:** 10.1007/s00018-023-04995-2

**Published:** 2023-10-28

**Authors:** Bin Lou, Guanghua Ma, Xiaopeng Yu, Feifei Lv, Fanjie Xu, Chengdi Sun, Yu Chen

**Affiliations:** 1 Department of Laboratory Medicine, The First Affiliated Hospital, Zhejiang University School of Medicine, Qingchun Road, Hangzhou, 310003 China; 2 Key Laboratory of Clinical In Vitro Diagnostic Techniques of Zhejiang Province, Hangzhou, 310003 China; 3 Institute of Laboratory Medicine, Zhejiang University, Hangzhou, 310003 China; 4 State Key Laboratory for Diagnosis and Treatment of Infectious Diseases, Zhejiang University, Hangzhou, 310003 China; 5 The Shengzhou Hospital of Traditional Chinese Medicine, Shaoxing, 312432 Zhejiang China

**Keywords:** HBV, OTUD5, Deubiquitinase, MAPK signal pathway, HNF4ɑ, HBV core/precore protein

## Abstract

Hepatitis B virus (HBV) infection is a major public health problem worldwide, causing nearly one million deaths annually. OTUD5 is a deubiquitinase associated with cancer development and innate immunity response. However, the regulatory mechanisms of OTUD5 underlying HBV replication need to be deeply elucidated. In the present investigation, we found that HBV induced significant up-regulation of OTUD5 protein in HBV-infected cells. Further study showed that OTUD5 interacted with HBV core/precore, removing their K48-linked ubiquitination chains and protecting their stability. Meanwhile, overexpression of OTUD5 could inhibit the MAPK pathway and then increase the expression of HNF4ɑ, and ERK1/2 signaling was required for OTUD5-mediated activation of HNF4α, promoting HBV replication. Together, these data indicate that OTUD5 could deubiquitinate HBV core protein degradation by its deubiquitinase function and promote HBV activity by up-regulating HNF4α expression via inhibition of the ERK1/2 pathway. These results might present a novel therapeutic strategy against HBV infection.

**Supplementary Information:**

The online version contains supplementary material available at 10.1007/s00018-023-04995-2.

## Introduction

Chronic viral disease hepatitis type B, which is caused by hepatitis B virus infection, is an important disease that threatens human health. An estimated 257 million people worldwide are chronically infected with HBV [1]. Carriers of HBV are at an increased risk of developing cirrhosis, hepatic decompensation, and ultimately hepatocellular carcinoma (HCC) [2, 3]. HBV belongs to the Hepadnaviridae family of small enveloped DNA viruses with a 3.2 kb partially double-stranded DNA genome. Once entered into blood circulation, HBV binds to its receptor, currently known as sodium taurocholate cotransporting polypeptide (NTCP), a multiple transmembrane transporter predominantly expressed in hepatic cells, resulting in viral entry [4]. Then, viral release the free nucleocapsid into the cytoplasm and translocated to the nucleus where relaxed circular DNA is released [5]. And the relaxed circular DNA is converted into a covalently closed circular DNA (cccDNA), which further interacts with histone proteins to form a minichromosome [6]. The HBV genome contains four overlapping open reading frames (S, C, P, and X). HBV cccDNA functions as a transcription template, encoding four major viral RNA species: pregenomic RNA (pgRNA, 3.5 kb), preS1 HBs RNA (2.4 kb), preS2/S HBs RNA (2.1 kb), and HBx mRNA encodes the HBV x protein (0.7 kb) [7]. Viral mRNAs are then transferred to the cytoplasm, functioning as templates for synthesizing viral proteins [8, 9].

Deubiquitinases in the OTU superfamily are originally identified from different organisms and affect antiviral immunity and virus proliferation [10–13]. OTUD5 is a member of OTU deubiquitinases which was reported to promote innate immunity through deubiquitinating and stabilizing STING Knockout of OTUD5 resulted in a faster turnover of STING and subsequently impaired type I IFN signaling following cytosolic DNA stimulation [14]. Meanwhile, OTUD5 can regulate the production of type I interferon via bounding tumor necrosis factor receptor-associated factor 3 (TRAF3), an adaptor protein essential for the IFN-I response [10]. However, the underlying mechanisms of OTUD5 for viral infection disease are ill defined, and the physiological functions of OTUD5 in HBV infection have remained largely unknown.

Hepatocyte nuclear factor 1α/4α (HNF1α/4α) are members of the nuclear hormone receptor family of transcription factors [15, 16] and play important roles in regulating the expression and replication of HBV by affecting most of the HBV regulatory elements or stimulating the transcription of HBV pgRNA. The mitogen-activated protein kinase (MAPK) pathway is known to regulate the expression of HNF4ɑ and lead to the suppression of HBV replication [17, 18]. This pathway inhibits HBV replication at the transcriptional level [19]. However, sources or agents that affect the MAPK pathway in hepatitis B infection disease are rarely studied.

In this study, we demonstrated that HBV induced significant upregulation of OTUD5 at the protein level, which displayed a stimulative effect on HBV transcription and replication. Further study showed that OTUD5 could stabilize HBV precore/core protein expression by hydrolyzing the ubiquitin chain at the K48 site. Another result showed that by activating the ERK1/2 signaling pathway, OTUD5 promoted the expression of HNF4α, a well-known activator of HBV core promoter which can promote the replication of HBV. Meanwhile, clinical HBV-infected patient serum was used to verify OTUD5 as a host marker to predict the seroconversion of HBeAg in patients with antiviral therapy.

## Materials and methods

### Cell culture and transfection

HEK293T, HepG2, and HepG2.2.15 cells were cultured in Dulbeccos Modified Eagle Medium (DMEM)culture medium supplemented with 10% heat-inactivated FBS and 100 U/ml penicillin, and 100 mg/ml streptomycin at 37 ℃ incubator containing 5% CO_2_. Mitogen-activated protein kinases (MAPK) inhibitors PD98059, SB203580, and SP600125 were purchased from ApexBio (ApexBio Technology, Houston, TX, USA). The plasmids of OTUD5-siRNA1 (5-GCAGTGTCCCAACAGGAATAC-3), OTUD5-siRNA2 (5-GGAGGAGTCATGGATTGAACA-3) and OTUD5-siRNA3 (5-GGAGGAGTCATGGATTGAACA-3), OTUD5-sgRNA1 (5-CTGTACTGGTACACCTCCACAGG-3), OTUD5-sgRNA2 (5-ACCGTGACTCCGGCGTCGTGGGG-3), and OTUD5-sgRNA3 (5-CGGCGCAGGCTACAACAGTGAGG-3) were transfected into cells using INVI DNA RNA Invigentech reagent (Invitrogen, Carlsbad, CA) following the manufacturer’s instructions.

### Patients

23 HBV-negative patients and 46 hepatitis B carriers were enrolled retrospectively from the First Affiliated Hospital, School of Medicine, Zhejiang University in this study to evaluate the expression of OTUD5 in the two cohorts. To further explore the predictor effect of OTUD5 in HBeAg seroconversion, the 62 CHB patient serum were collected during 2013–2016 [3]. This project was approved by the 1964 Helsinki Declaration and its later amendments, and prior informed consent was obtained from the enrolled patients.

### Antibodies

Antibodies against p38 (#8690), p-P38 (#4511), ERK1/2 (#4695), p-ERK1/2 (#4376), JNK (#9212), p-JNK (#9255), HNF1α (#89670), HNF4α (#3113) were obtained from Cell Signaling Technology (Cell Signaling Technology, Inc., Danvers, MA, USA). Antibodies against OTUD5 (ab254742), HBc (ab8638), HBx (ab2741), GAPDH (ab181602), Ubiquitin linkage-specific K48 (ab140601), Ubiquitin linkage-specific K63 (ab179434) were obtained from Abcam (Abcam, Cambridge, UK).

### Quantitative RT-PCR (qRT-PCR)

Total RNA was extracted from culture cells by Trizol reagent and reverse-transcribed into cDNA, followed by quantitative real-time PCR using Thermo Fisher ABI7500 (ABI Laboratories, USA) and SYBR green system (Vazyme, Nanjing, China). The following primers were used for PCR: OTUD5 forward 5-CGCTATGTGGATACGCTGCTTTA-3 and OTUD5 reverse 5-GCAACCAGGATTTATACAAGGAGGA-3; GAPDH forward 5-GTCTCCTCTGACTTCAACAGCG-3 and GAPDH reverse 5-ACCACCCTGTTGCTGTAGCCAA-3; HBV RNA forward 5-GCACTTCGCTTCACCTCTGC-3 and HBV RNA reverse 5-CTCAAGGTCGGTCGTTGACA-3; HBV pg RNA forward 5-TGTTCAAGCCTCCAAGCT-3 and HBV pg RNA reverse 5-GGAAAGAAGTCAGAAGGCAA-3. The relative expression of target genes was calculated by the 2^−ΔΔCt^ method, and GAPDH was used as an internal control.

### Quantification of HBsAg, HBeAg, and HBV DNA in the culture medium

Cells were seeded in 6-well plates at a density of 8 × 10^8^ cells/well in DMEM containing 10% FBS. After 12 h of incubation, the cells were treated with various concentrations of OTUD5-sh and OTUD5-overexpression plasmid for 2 d. The HBsAg and HBeAg in the culture medium were measured using chemiluminescence microparticle immunoassay (CMIA) Abbott Architect I4000 automated analyzer (Abbott Laboratories, Chicago, IL, USA). The HBV DNA levels were measured using the ABI7500 quantitative Real-Time PCR System (ABI Laboratories, USA), and the PCR kits were obtained from Qiagen (Hilden, Germany).

### Co-immunoprecipitation

Cells were lysed 48 h after transfection of expression plasmids using 50 mM Tris–HCl, pH 8.0, 150 mM NaCl, and 1% NP-40 containing cocktail inhibitors. For immunoprecipitation, lysates were incubated overnight with anti-FLAG^®^ M2 magnetic beads (Sigma, USA) or anti-Myc magnetic beads (CST, USA).

### Western blot

Cell lysates were separated by sodium dodecyl sulfate–polyacrylamide gel electrophoresis (SDS-PAGE) and transferred into a polyvinyl difluoride (PVDF) membrane. The PVDF membranes were incubated with indicated primary antibodies at 4 °C overnight. The next day, membranes were incubated with secondary antibodies at room temperature for 1 h and observed using the enhanced chemiluminescence (ECL) detection reagents.

### Immunohistochemistry

HBV core protein and OTUD5 in HBV patient liver tissues were examined by immunohistochemical. Briefly, paraffin-embedded liver tissue sections were treated with 3% hydrogen peroxide and blocked with 5% bovine serum albumin. The sections were then incubated with anti-HBc and anti-OTUD5, biotinylated secondary antibody, and avidin–biotin complex (ABC). The staining was developed with diaminobenzidine (DAB) solution and counterstained with hematoxylin.

### In vitro ubiquitination assay

HEK293T cells were transfected with HBV precore/core plasmid and OTUD5 knockdown or overexpression plasmids. After 48 h, cells were treated with MG132 (10 μM) for 8 h. Then, cells were harvested and ubiquitinated hepatitis B precore or hepatitis B core was purified from the cell extracts and immunoprecipitation with Anti-Flag M2 Magnetic Beads (Sigma, USA), and cell lysates and precipitated samples were analyzed by immunoblotting with the related antibodies.

### Immunofluorescent confocal microscopy assay

HEK293T cells were seeded in 6-well plates at a density of 8 × 10^8^ cells/well in DMEM containing 10% FBS. After 12 h of incubation, the cells were treated with HBV precore/core or HBx plasmid with green fluorescent protein (GFP) expression tag and OTUD5 with red fluorescent protein (RFP) expression tag plasmid for 2 days. Then, the cells were fixed with 4% paraformaldehyde for 10 min followed by PBS wash three times, then stained with DAPI (Beyotime Biotechnology) and examined under a confocal microscope (TCS SP8; Leica Microsystems Inc., Buffalo Grove, IL, USA).

### Statistical analysis

Statistical significance for all experiments was determined using Students *t* test or the Mann–Whitney rank sum test (GraphPad Prism 9 software). Error bars are reported as standard errors of the means, and significance was assigned for *P* values of *P* < 0.05. *P < 0.05, **P < 0.01, ***P < 0.001.

## Results

### OTUD5 expression in HBV-infected cell line and HBV-infected patients

**Fig. 1:**
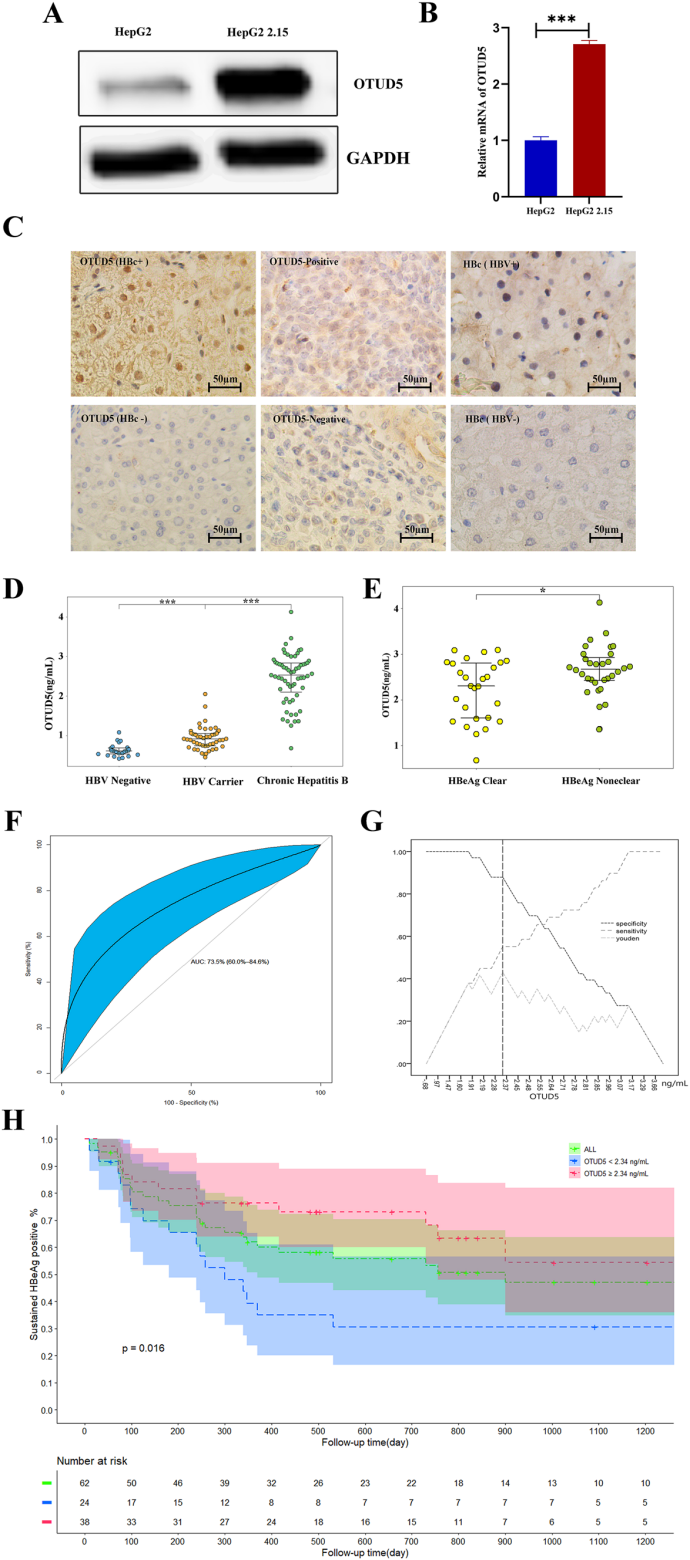
The effect of HBV infection on the expression level of OTUD5. Western blot results showed elevated expression of OTUD5 in HepG2.2.15 cells compared with HepG2 cells (**A**). Levels of OTUD5 mRNA in HepG2.2.15 cells compared with HepG2 cells (**B**). Expression of OTUD5 in liver tissues based on IHC. (Left) Overexpression OTUD5 was observed in the cytoplasm in chronic hepatitis B liver tissues compared with HBV-negative liver tissues. (Middle) OTUD5 expression as positive control in the human carcinoma of the rectum tissues compared with normal rectum tissues. (Right) HBc expression in HBV-positive patients compared with HBV-negative patients (scale bar 50 μm) (**C**). OTUD5 levels in HBV negative cohort, HBV carriers and chronic hepatitis B patients (**D**). OTUD5 concentration at baseline between HBeAg seroconversion group and HBeAg none seroconversion group (**E**). ROC curve of OTUD5 as a host marker to predict HBeAg seroconversion in the follow-up period (**F**, **G**). Analysis of HBeAg seroconversion rate using the Kaplan–Meier method during the follow-up (**H**) (*P < 0.05, ***P < 0.001)

To examine the effect of HBV on the OTUD5 expression, the OTUD5 protein and mRNA levels were detected in HepG2.2.15 which stably produced HBV virus and HepG2 cell line, the OTUD5 protein and OTUD5 mRNA were significantly higher in HepG2.2.15 (Fig. 1A,B). To further verify the OTUD5 expression in HBV-infected patients, IHC was performed in HBV-positive and HBV-negative livers. Overexpression OTUD5 was observed in the cytoplasm in chronic hepatitis B liver tissues compared with HBV-negative liver tissues (Fig. 1C).

In this study, we also enrolled 23 HBV-negative patients, 46 Hepatitis B virus carriers and 62 chronic hepatitis B patients, and we found the concentration of OTUD5 in the serum of HBV carriers was strongly higher than in healthy people. Interestingly, the titer of OTUD5 in CHB patients was significantly higher than HBV carriers (*P* < 0.05) (Fig. 1D).

Meanwhile, 62 CHB patients with antiviral therapy in the follow-up period were analyzed. In 32 patients who achieved HBeAg seroconversion group, the OTUD5 titer was lower than the 30 patients without HBeAg seroconversion (*P* < 0.05) (Fig. 1E), which meant the HBeAg positive CHB patients with lower levels of OTUD5 would be more likely to achieve HBeAg clear after antiviral treatment. Meanwhile, the ROC curve and Kaplan–Meier analyses were performed in two groups, and results showed that OTUD5 was a satisfied predictor of HBeAg seroconversion and the concentration of 2.34 ng/mL cut-off value for response in ROC and Kaplan–Meier curve analysis (Fig. 1F–H).

### OTUD5 promoted HBV transcription and replication

**Fig. 2:**
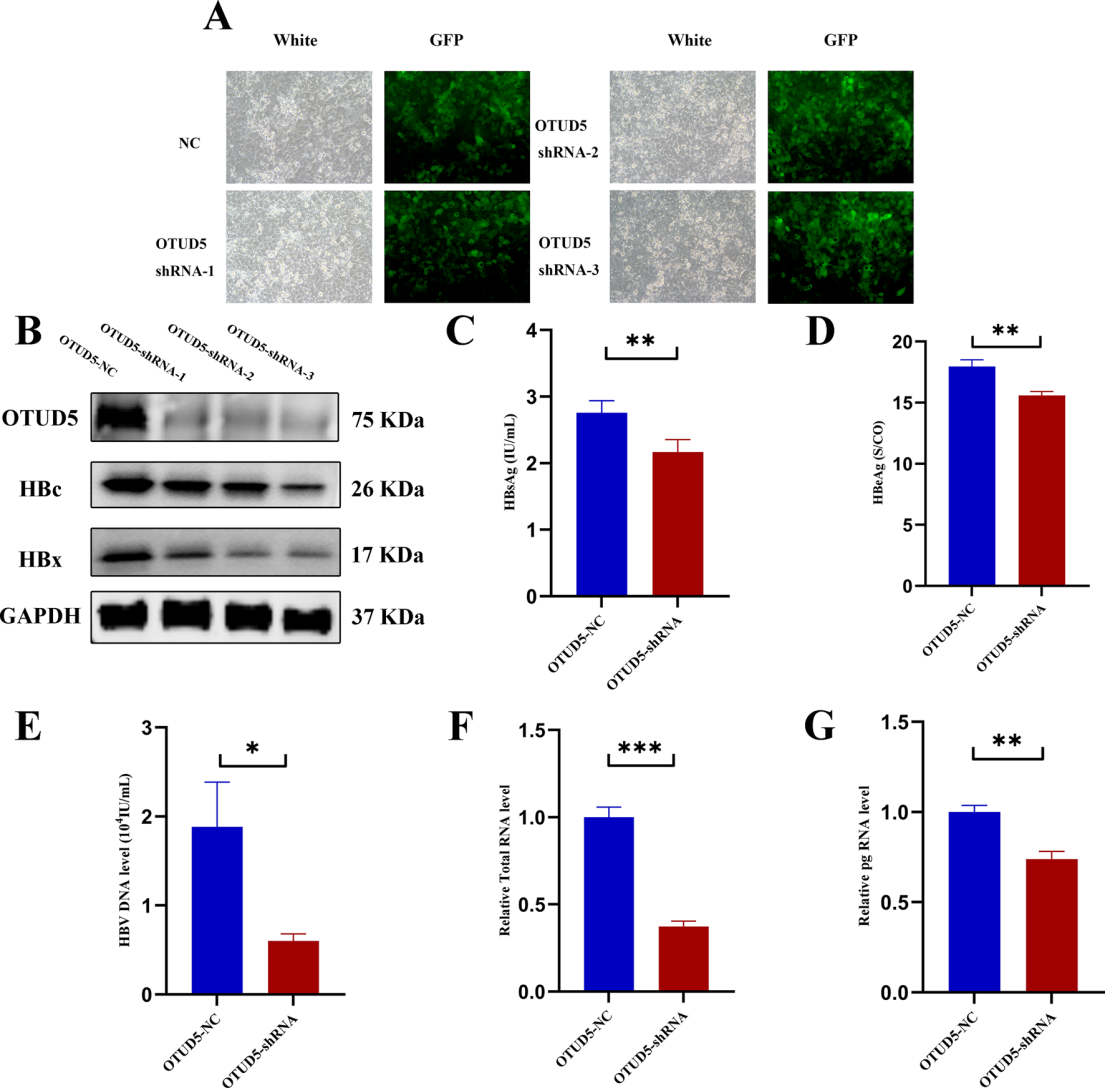
Knockdown of OTUD5 inhibited HBV transcription and replication in HepG2.2.15 cell lines. HepG2.2.15 cells were infected with OTUD5-sh lentivirus plasmid and mock vector as a negative control with GFP (**A**). Western blot results showed that the HBc and HBx proteins were inhibited when the OTUD5-sh lentivirus plasmid was transfected into HepG2.2.15 cells (**B**). The concentrations of HBsAg, HBeAg and HBV DNA were decreased in OTUD5-sh compared with negative control in culture supernatants (**C**–**E**). The levels of pg RNA and HBV total RNA were both significantly decreased in HepG2.2.15 cells with OTUD5 knockdown (**F**–**G**) (*P < 0.05, **P < 0.01, ***P < 0.001)

The above results showed OTUD5 was elevated in infected patients and cell line. We then investigated whether OTUD5 could affect HBV replication. The OTUD5 knockdown plasmid with GFP (Green Fluorescent Protein) tag or knockout plasmid was constructed by siRNA or CRISPR–cas9 technique, and then these plasmids were transfected into HepG2.2.15 cells. Empty vectors were used as the negative control. The knockdown and knockout efficiencies of OTUD5 were confirmed by Western blot (Fig. 2B and S1A). Knockdown or knockout of OTUD5 significantly downregulated the replication and transcription of HBV, as demonstrated by the increased levels of HBsAg (Fig. 2C and S1B), HBeAg (Fig. 2D and S1C) and HBV-DNA (Fig. 2E and S1D) in culture supernatants and total HBV RNA (Fig. 2F and S1E) and HBV pg RNA (Fig. 2G and S1F) in HepG2.2.15 cells.

**Fig. 3:**
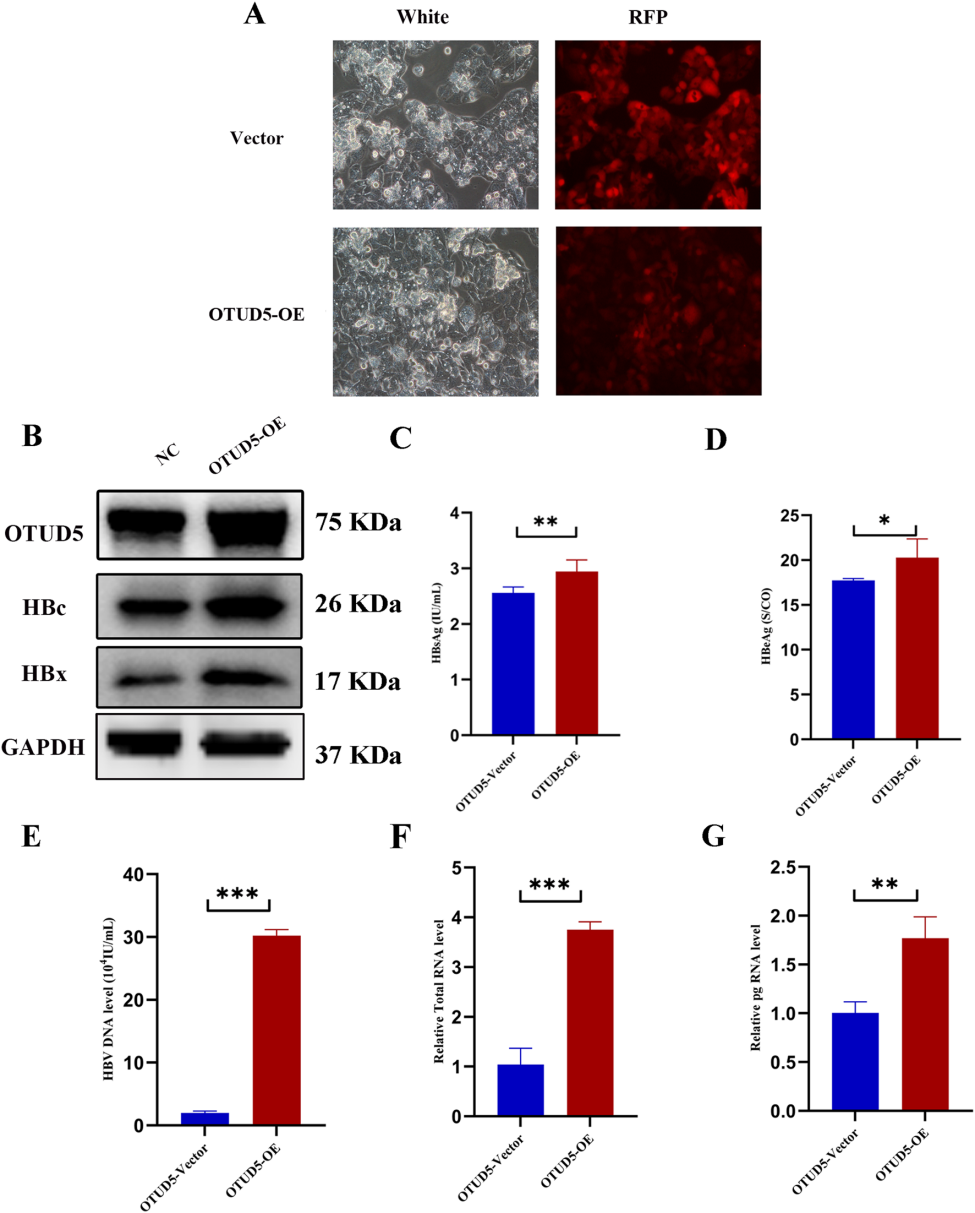
Overexpression of OTUD5 promoted HBV replication and transcription. HepG2.2.15 cells were infected with OTUD5 lentivirus plasmid and mock vector as negative control with RFP (**A**). Western blot results showed that the HBc and HBx proteins were elevated when the OTUD5 lentivirus plasmid was transfected into HepG2.2.15 cells (**B**). The concentrations of HBsAg, HBeAg and HBV DNA were increased in OTUD5 overexpression compared with the mock vector in culture supernatants (**C**–**E**). The levels of pg RNA and HBV total RNA were both significantly elevated in HepG2.2.15 cells with OTUD5 overexpression (**F**, **G**) (*P < 0.05, **P < 0.01, ***P < 0.001)

To further confirm the promotion effect of OTUD5 on HBV transcription and replication, the OTUD5 lentivirus expression plasmid with RFP (Red Fluorescent Protein) or empty vector with RFP was transfected into HepG2.2.15 cells. Compared with decreased expression of OTUD5, inverse results were observed in overexpression of OTUD5 (Fig. 3). Meanwhile, HBc protein and mRNA level were analyzed in the same experiment, in control, OTUD5 OE, KD and KO group. The results were consistent with the above experiment (Fig. S2). In conclusion, these results displayed that OTUD5 was an important promotor involved in HBV transcription and replication in vitro.

### OTUD5 interacted with HBV precore/core proteins

**Fig. 4:**
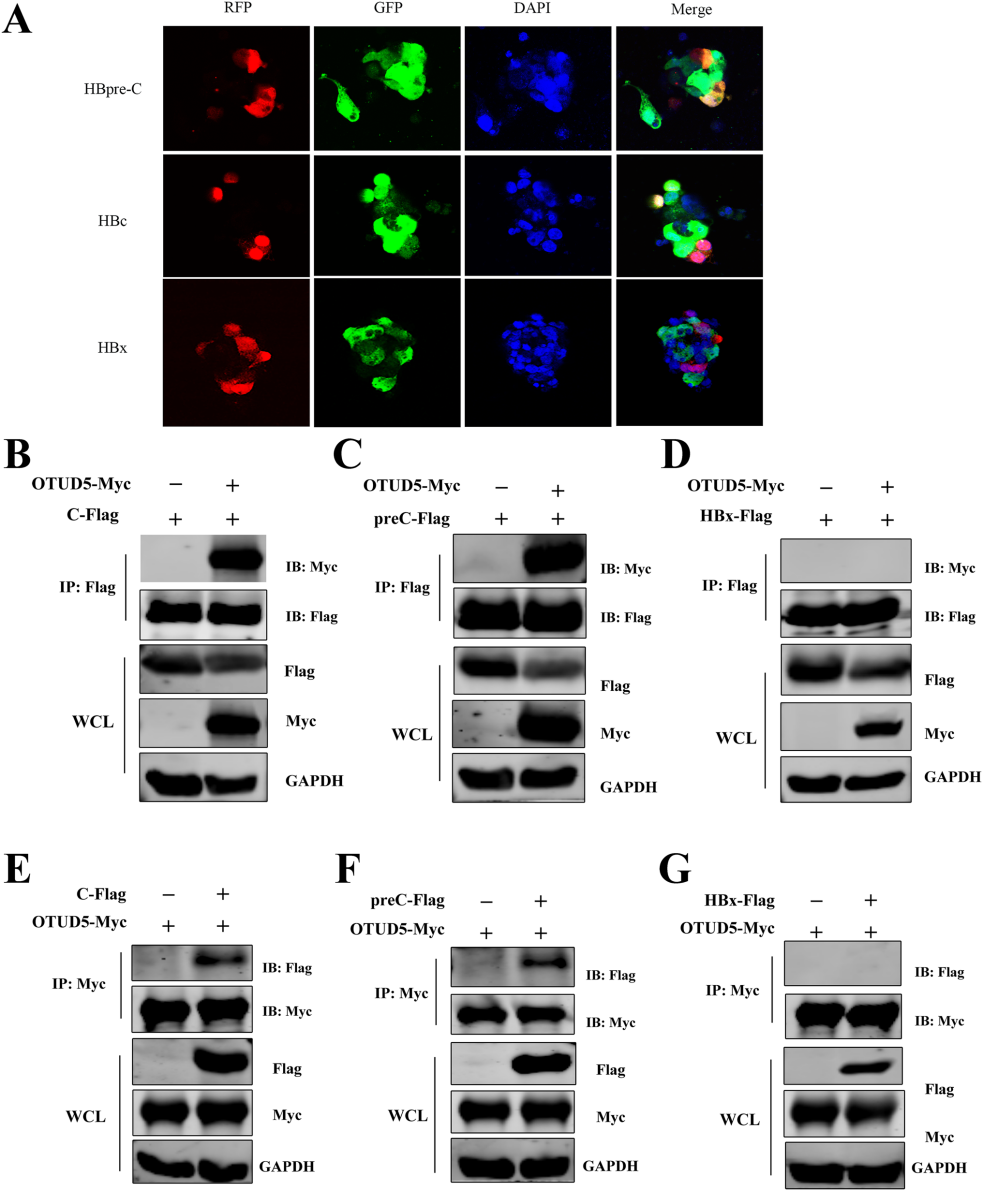
OTUD5 interacted with hepatitis B precore/core proteins. OTUD5 plasmid with RFP and hepatitis B precore, core and x plasmids with GFP were cotransfected into HEK293T cells, and the interactions between OTUD5 and hepatitis B precore, core and x proteins were observed using confocal microscopy (**A**). OTUD5 plasmid with myc tag and hepatitis B precore, core and x plasmids with flag tag were co-transfected into HEK293T cells, and the interactions between OTUD5 and hepatitis B precore, core and x proteins were analyzed using anti-flag magnetic beads (**B**–**D**). OTUD5 plasmid with myc tag and hepatitis B precore, core and x plasmids with flag tag were co-transfected into HEK293T cells. The interactions between OTUD5 and hepatitis B precore, core and x proteins were analyzed using anti-myc magnetic beads (**E**–**G**)

We hypothesized that OTUD5 could promote HBV viral transcription and replication through interactions with individual viral proteins. In the present study, the OTUD5 plasmid with GFP tag and HBV structure protein plasmids with RFP tag were co-transfected into HEK293T cells, and confocal microscopy assay was used to explore the interaction between OTUD5 and HBV proteins, the results showed HBV precore and core proteins both had colocalization with OTUD5 (Fig. 4A).

Meanwhile, we used a coimmunoprecipitation assay to screen HBV structure proteins for interaction with OTUD5. In the coimmunoprecipitation assay, the OTUD5 plasmid with Myc tag and HBV structure protein plasmids with Flag tag were transfected into HEK293T cell, Flag tag magic beads (Fig. 4B–D) and Myc tag magic beads (Fig. 4E–G) were used to analyze the interaction of OTUD5 and HBV proteins. Furthermore, endogenous HBV core and OTUD5 interaction experiments were also performed in coimmunoprecipitation and colocalization assays (Fig. 3S). These results from confocal and coimmunoprecipitation indicated HBV core and precore proteins both had interaction with OTUD5, while HBx protein did not display interaction with OTUD5.

### OTUD5 stabilized HBV core proteins via deubiquitination

**Fig. 5:**
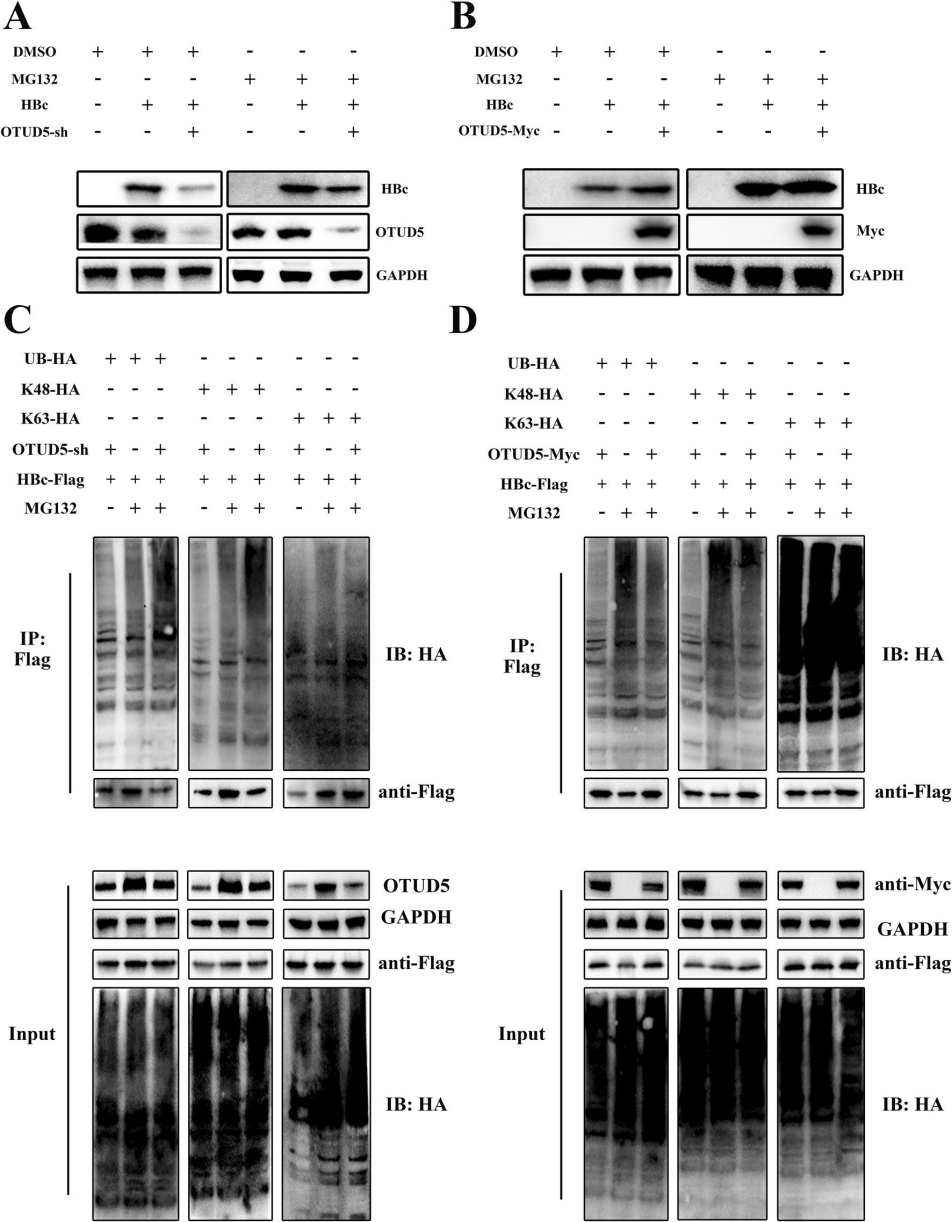
OTUD5 inhibited the hepatitis B core protein degradation through the proteasome pathway. Hepatitis B core plasmids with flag tag and OTUD5-sh or OTUD5 overexpression plasmid with myc tag were co-transfected into HEK293T cells. After transfection, the cells were treated with MG132 or DMSO for 8 h before harvest, then cell lysates were collected and subjected to Western blot (**A**, **B**). Hepatitis B core plasmid with flag tag, ubiquitin plasmids with HA tag and OTUD5 knockdown or overexpression plasmid were co-transfected into HEK293T cells. After transfection, the cells were treated with MG132 or DMSO for 8 h. Lysates immunoprecipitation and whole cell lysates analysis of the deubiquitination of HBc were performed by Western blot (**C**, **D**)

HBV core is an indispensable replication factor in the HBV life cycle, which regulates cccDNA formation, a critical step in the establishment and persistence of HBV infection [20]. The above results showed that HBV core proteins interacted with OTUD5. Due to the deubiquitination function of OTUD5, we asked if the induction of OTUD5 by HBV could display its deubiquitinase character on HBV core proteins. To answer this question, MG132 was used to determine that OTUD5 could prevent HBc degradation by deubiquitination function through the ubiquitin-dependent proteasome pathway (Fig. 5A,B). Then, OTUD5 protein expression plasmid or knockdown plasmid were co-transfected with HBV core protein expression plasmid and ubiquitin plasmids into HEK293T cells. Using Flag tag beads, the coimmunoprecipitation experiment was performed to analyze the ubiquitination status of HBV core. The results showed that the knockdown of OTUD5 gene could promote the ubiquitination of HBV core proteins at the K48 site (Fig. 5C). The opposite results were observed in upregulated OTUD5 in HEK293T cells by overexpression technique (Fig. 5D). Meanwhile, the antibodies to endogenous ubiquitin were used to analyze endogenous ubiquitin in the status of HBV precore/core plasmids were co-transfected with OTUD5 overexpression (Fig. S4A–C) or OTUD5-sh plasmids (Fig. S4D–F).

The HBV precore protein, translated from the precore mRNA, had the same sequence as HBc and only had an N-terminal 29 amino acid (aa) extension [21]. we have proved HBV precore/core proteins both interacted with OTUD5, excluding the interaction position on the N-terminal 29 amino acids. DUBs usually prevent substrate degradation by deubiquitination through the ubiquitin-dependent proteasome pathway [22]. Therefore, we selected the HBV core protein to confirm further the protective effect on HBc via the deubiquitination character of OTUD5. The HBc and different titer of OTUD5 expression plasmid or different titer of OTUD5 knockdown plasmid were co-transfected into HEK293T cells. These results showed that OTUD5 strongly inhibited HBc degradation in vitro (Fig. S4G, H).

### OTUD5 promoted HBV replication via upregulating HNF4α

**Fig. 6:**
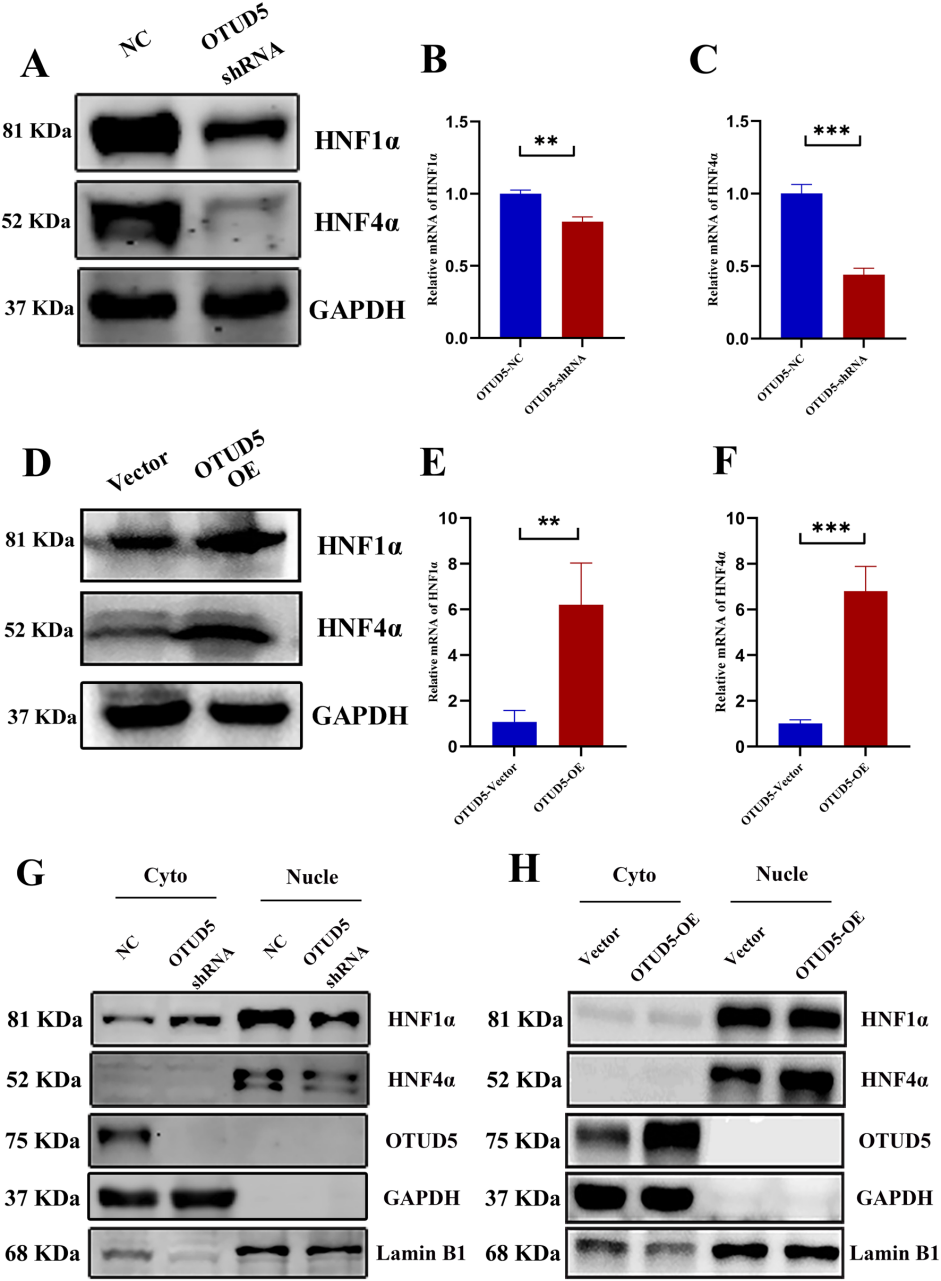
OTUD5 promoted the levels of HNF1ɑ and HNF4ɑ. OTUD5 downregulation in HepG2.2.15 cells decreased the expression of HNF1ɑ and HNF4ɑ (**A**–**C**). Overexpression of OTUD5 in HepG2.2.15 cells increased HNF1ɑ and HNF4ɑ (**D**–**F**). Knockdown of OTUD5 decreased the distribution of HNF1ɑ and HNF4ɑ in nuclear (**G**), overexpression of OTUD5 increased the distribution of HNF4ɑ, while the HNF1ɑ in nuclear did not increase after over-expressing OTUD5 compared with mock vector in HepG2.2.15 cells (**H**)

Studies have proved liver-enriched transcription factors (LETFs) such as CEBP, NR2F1, NR1H4, HNF1ɑ and HNF4ɑ play a crucial role in regulating HBV replication and transcription [23]. The above results showed OTUD5 displayed a significant promoting effect on HBV replication and transplantation, and we further invested the role of OTUD5 on the expression of LETFs. The data of PCR showed that knockdown of OTUD5 significantly downregulated the mRNA of HNF1ɑ and HNF4ɑ (Fig. 6B,C). Conversely, the overexpression of OTUD5 could upregulate the mRNA of HNF1ɑ and HNF4ɑ (Fig. 6E–F). Western blot analysis was used to confirm further the promoting effect of OTUD5 on HNF1ɑ and HNF4ɑ, and the results were consistent with the PCR results (Fig. 6A,D). To investigate whether the level of HNF1ɑ and HNF4ɑ between nuclear and cytoplasm were different, we analyzed the distribution and variation of HNF1α and HNF4α in cytoplasm and nucleus. When the OTUD5 gene was knocked down in HepG2.2.15, HNF1α and HNF4α expression in the nucleus decreased (Fig. 6G). However, after OTUD5 overexpression, only the level of HNF4α in the nucleus increased (Fig. 6H).

### MAPK was required for OTUD5-mediated promotion of HBV replication via HNF4α

**Fig. 7:**
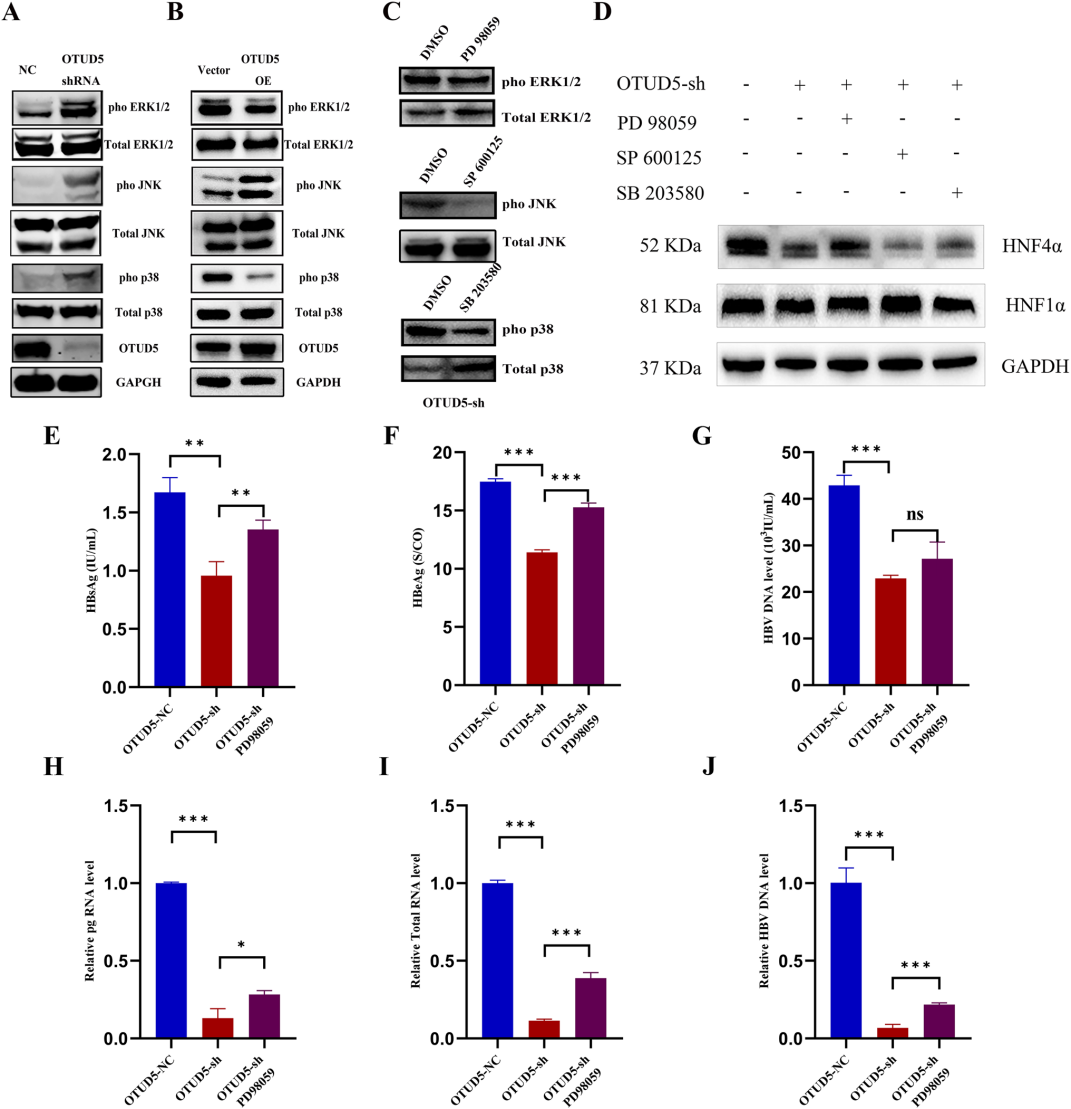
ERK1/2 signaling was involved in the OTUD5-mediated promotion of HNF4α and HBV. HepG2.2.15 cells were transfected with OTUD5 overexpression lentivirus plasmid or OTUD5 knockdown lentivirus plasmid, and cell lysates were collected and subjected to Western blot using antibodies against ERK1/2, p-ERK1/2, JNK, p-JNK, p38, p-p38, OTUD5, or GAPDH (**A**, **B**). HepG2.2.15 cells were transfected with OTUD5 knockdown lentivirus plasmid, 36 h post-transfection, cells were treated with ERK-specific pharmacological inhibitor PD98059 (10 μM), JNK-specific pharmacological inhibitor SP600125 (10 μM) and p38-specific pharmacological inhibitor SB203580 (10 μM) or DMSO for another 24 h, cells were lysed and subjected to Western blot using antibodies against ERK1/2, p-ERK1/2, JNK, p-JNK, p38, p-p38 (**C**). HepG2.2.15 cells were transfected with OTUD5 knockdown lentivirus plasmid and mock vector as negative control, 36 h post-transfection, cells were treated with PD98059 (10 μM), SP600125 (10 μM) and SB203580 (10 μM) for another 24 h, cells were lysed and analyzed by Western blot using antibodies against HNF4ɑ, HNF1ɑ or GAPDH antibodies (**D**). HepG2.2.15 cells were transfected with OTUD5 knockdown lentivirus plasmid and mock vector as the negative control, 36 h post-transfection, cells were treated with PD98059 (10 μM) or DMSO for another 24 h, HBsAg, HBeAg and HBV DNA were tested in culture supernatant fluid (**E**–**G**), the expression of pgRNA, total HBV RNA and HBV DNA in cells were analyzed by PCR (**H**–**J**)

MAPK signaling pathways were important in controlling HNF1α and HNF4α expression [24–26]. The above results implicated OTUD5 was involved in the expression of HNF4α. To investigate the effect of OTUD5 on the MAPK signaling pathways, the pivotal proteins of MAPK were analyzed by Western blot in OTUD5 knockdown or overexpression in HepG2.2.15 cells. The results showed OTUD5 knockdown significantly enhanced the phosphorylation of ERK1/2, p38 and JNK (Fig. 7A), while after overexpression of OTUD5, the inverse results were only observed in ERK1/2 and p38 pathways (Fig. 7B). To further investigate the role of ERK1/2 and p38 signaling in OTUD5-mediated promotion of HNF4ɑ or HNF1ɑ, we treated the OTUD5-sh HepG2.2.15 cells with ERK1/2-specific pharmacological inhibitor (PD98059), JNK-specific pharmacological inhibitor (SP600125) and p38-specific pharmacological inhibitor (SB203580), and then tested the effect of OTUD5 on the protein level of HNF1α and HNF4α. As expected, PD98059, SP600125, and SB203580 could efficiently inhibit the activation of ERK1/2, JNK and p38, respectively (Fig. 7C). However, the inhibition of ERK1/2 but not JNK and p38 MAPK pathways enhanced OTUD5-mediated promotion of HNF4α (Fig. 7D).

To further confirm the role of ERK1/2-specific pharmacological inhibitor for HBV replication and transcription, HepG2.2.15 cells, OTUD5-sh HepG2.2.15 cells and OTUD5-sh HepG2.2.15 cells treated with PD98059 cells were established and cultured, then the cells and culture supernatant fluid were collected. HBsAg, HBeAg and HBV DNA were tested in culture supernatant fluid. Compared with none PD98059 treated OTUD5-sh HepG2.2.15 cells, the HBsAg and HBeAg were rising significantly (Fig. 7E,F), and the upward trend of HBV DNA was also observed even though the difference was not markedly (Fig. 7G). The levels of HBV pgRNA, Total RNA, and DNA in cells also rose significantly (Fig. 7H–J).

## Discussion

In this study, we demonstrated that deubiquitinase OTUD5 is a positive regulator of the hepatitis B virus, participating in regulating HBV transcription and replication (Fig. 1A–D). OTUD5, as one member of the OUT subfamily of DUBs, had reported to play critical roles in multiple cellular processes. OTUD5 was also reported to regulate DNA damage response at damaged chromatin in a FACT-dependent transcription [27]. However, the mechanism of OTUD5 in HBV replication and transcription has yet to be studied. In this study, we showed the important roles and underlying mechanisms of OTUD5 in HBV replication and transcription, highlighting the therapeutic targets for HBV treatment.

We demonstrated deubiquitinase OTUD5 interacted with HBV precore/core proteins (Fig. 4). Posttranslational regulation of hepatitis B virus structural proteins had been an important cellular process during HBV infection. For example, TRIM21 inhibited HBV replication through ubiquitin-mediated proteasomal degradation of HBx [28], and NEDD4 prohibited HBV infection by mediating K48-linked degradation of HBx and inhibiting HBV-associated HCC [29]. In addition, the deubiquitinating enzyme VCPIP1 binds to HBx protein and promotes stabilization of HBx in a ubiquitin-independent manner by recruiting the PSMC3 in vivo [30]. Therefore, ubiquitin-mediated or deubiquitin-mediated HBV proteins are a critical regulatory step during HBV infections. Considering the deubiquitinase function of OTUD5, a ubiquitination assay in vitro was performed in this study. We demonstrated that overexpression of OTUD5 could remove the K48-linked ubiquitination of HBV precore/core proteins. At the same time, the knockdown of OTUD5 reversed that effect (Fig. 5). Deubiquitinase OTUD5 on HBV core protein to protect its stabilization was further verified in our study (Fig. S4G, H). HNF1α/HNF4α had been shown as important components during HBV infection. In the present study, we discovered that overexpression of OTUD5 promoted HNF1α/HNF4α protein and mRNA expression while down-expression of OTUD5 inhibited HNF1α/HNF4α protein and mRNA expression in hepG2 2.15 cells (Fig. 6A–E). During HBV infection, nuclear transcriptional factors HNF1α/HNF4α could shuttle from cytoplasm to nucleus to bind to the proximal regulatory element of HBc promoter, thus producing 3.5 kb pgRNA production [31]. In hepG2.2.15 cells, we also found knockdown of OTUD5 could inhibit the translocation of HNF1α/HNF4α into nuclear. However, the overexpression of OTUD5 showed a diverse result in HNF4α, and the change in HNF1α was insignificant (Fig. 6G,H).

The activation of HNF1α/HNF4α required MAPK signaling participation. As reported, p38-p42/p44-JNK MAPKs were involved in HNF1α/HNF4α expression and cytoplasm-nuclear translocation [32]. In this study, we proved OTUD5 could downregulate the expression of phosphorylation ERK1/2 in hepG2.2.15 cells, and the results illustrated that OTUD5 could promote the expression of HNF4α via inhibiting ERK1/2 of MAPK pathway (Fig. 7A–D). For instance, studies showed that HNF1α/HNF4α had played an important role in HBV release and regulation of HNF1α/HNF4α expression had been targeted as one of the anti-HBV approaches [33–35]. Therefore, we next evaluated the promotion effect on HBV replication and transcription required the ERK1/2 pathway induced by OTUD5. Using an inhibitor of ERK1/2, we demonstrated OTUD5 inhibited ERK1/2 MAPK signaling pathway to accumulate HNF4α expression to benefit HBV replication.

**Fig. 8:**
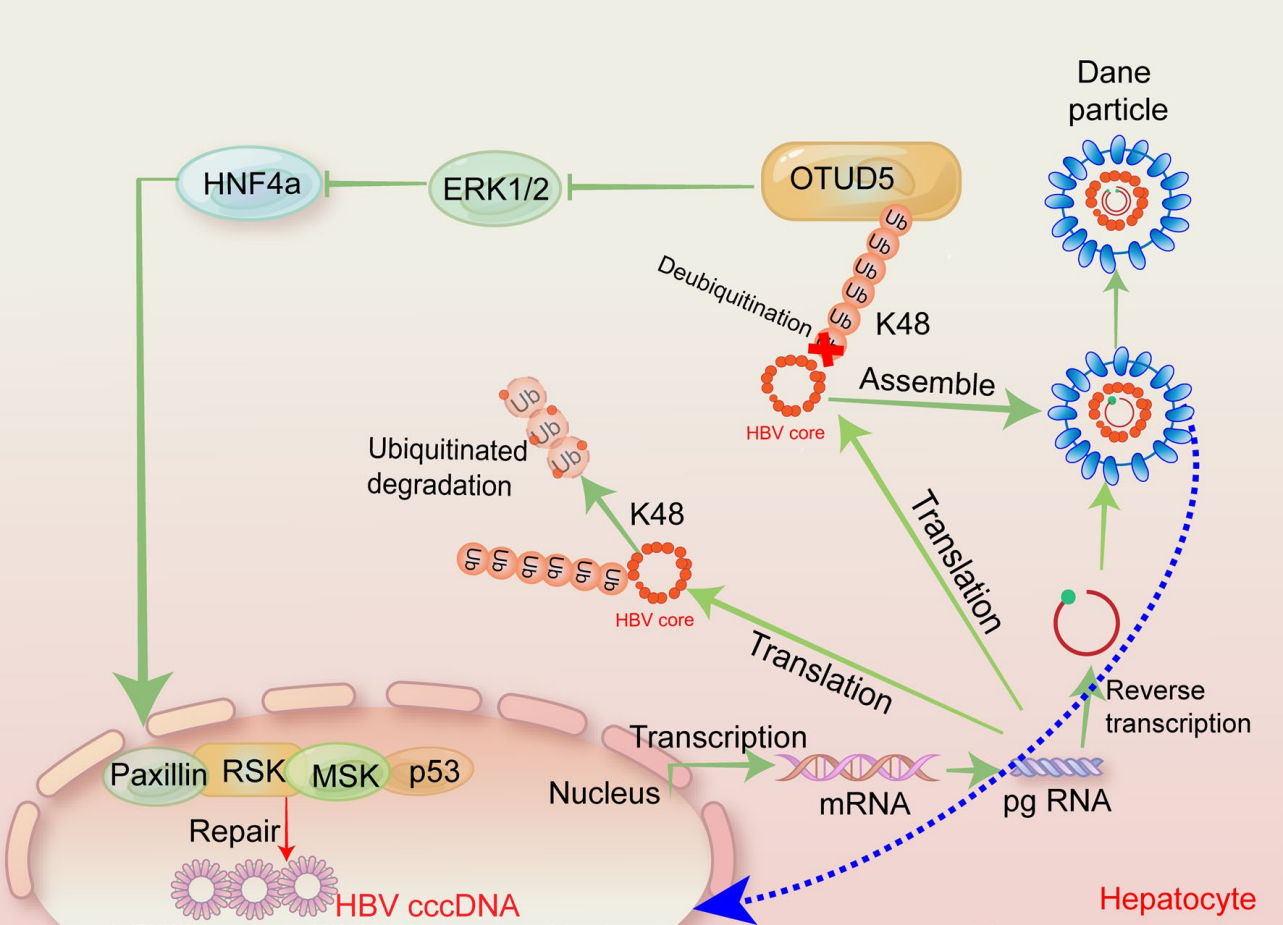
Model depicting the crosstalk between MAPK signal pathway and OTUD5 and the deubiquitination activity of OTUD5 on HBc protein

In this study, the healthy cohort, HBV carriers and chronic hepatitis B patients were enrolled, the expression OTUD5 in different groups was analyzed, and significant differences of OTUD5 were observed between the HBV-negative cohort and HBV positive carriers, meaning the expression of OTUD5 in human serum was promoted by infecting hepatitis B virus. Interestingly, compared with positive carriers, the OTUD5 serum concentration in chronic hepatitis B patients was remarkably higher (Fig. 8).

Based on the above results, OTUD5 was an important deubiquitinase for stabilizing HBV precore/core proteins that are highly relevant to HBeAg. Therefore, we hypothesized whether OTUD5 correlated with HBeAg seroconversion under anti-viral treatment in CHB patients. To identify whether OTUD5 has a predictive characteristic in HBeAg seroconversion, the OTUD5 titer of chronic hepatitis B patients at different timepoints was analyzed between HBeAg seroconversion and HBeAg none seroconversion groups, and the significant difference was concluded between the two groups. Using the ROC curve and Kaplan–Meier method, OTUD5 was demonstrated as an interesting biomarker for predicting HBeAg seroconversion.

In summary, we revealed that deubiquitinase OTUD5 could positively regulate HBV transcription and replication in HepG2.2.15 cells by deubiquitinating the K48-mediated ubiquitination of hepatitis B precore/core components to promote HBV infection. In addition, we also proved that OTUD5 could regulate HNF1α/HNF4α cytoplasm-nuclear translocation to affect HBV release. This study not only elucidates the mechanistic study of HBV replication but also provides novel therapeutic strategies to combat HBV infection.

### Supplementary Information

Below is the link to the electronic supplementary material.

Knockout of OTUD5 inhibited HBV transcription and replication in HepG2. 2.15 cell lines. HepG2.2.15 cells were infected with OTUD5-sg plasmid using CRISPR–cas9 technology and mock vector as the negative control, and Western blot results showed the knockout effect of OTUD5 (A). The concentration of HBsAg and HBeAg was decreased in OTUD5-sg#2 compared with a negative control in supernatant (B–C, F). The expression of pg RNA and HBV total RNA significantly decreased in HepG2.2.15 OTUD5-sg#2 cells compared with negative control (D-E) (TIF 360 KB)

Endogenous HBV core at protein and mRNA level in the same experiment, in control, OTUD5 OE, KD and KO. HepG2.2.15 cells were infected with OTUD5-control, OTUD5-overexpression, OTUD5-sh and OTUD5-sg plasmids. Western blot results showed the protein levels of OTUD5 and HBc, PCR result showed the relative expression of HBc mRNA (TIF 938 KB)

Interaction experiments of endogenous HBV core and OTUD5. Endogenous HBV core and OTUD5 interaction experiments were performed in coimmunoprecipitation and colocalization assays. HepG2.2.15 cells were harvested after treatment with MG132 for 8 h. Cellular lysates were subjected to IP with anti-HBc and IB with anti-OTUD5 (A). Confocal microscopy analysis of colocalization of endogenous OTUD5 with HBc. HepG2.2.15 cells were incubated with anti-HBc and anti-OTUD5 overnight, then incubated with a secondary antibody conjugated to Alexa Fluor 637 or Alexa Fluor 488 for fluorescent staining (B) (TIF 6957 KB)

OTUD5 inhibited the hepatitis B precore/core proteins degradation through the proteasome pathway in a ubiquitin-independent manner. Hepatitis B precore/core plasmids with flag tag and OTUD5 overexpression plasmid with myc tag were co-transfected into HEK293T cells. After transfection, the cells were treated with MG132 for 8 h, lysates immunoprecipitation and whole cell lysates analysis of the deubiquitination of HBc were performed by Western blot (A-C). Hepatitis B precore/core plasmids with flag tag and OTUD5 knockdown plasmid were co-transfected into HEK293T cells. After transfection, the cells were treated with MG132 for 8 h, lysates immunoprecipitation and whole cell lysates analysis of the deubiquitination of HBc were performed by Western blot (D-F). HEK293T cells were co-transfected with HBc-Flag and different doses of OTUD5 overexpression or OTUD5 knockdown plasmids. After 72 h, the cells were lysed and HBc protein levels were analyzed by Western blot using the anti-flag antibody (G–H) (TIF 5399 KB)

## Data Availability

The data supporting the findings of this study are found in the article and the supplementary material. The corresponding author will make all relevant raw data available upon reasonable request.
