# Supplementary figures and images for: Deubiquitinase OTUD5 promotes hepatitis B virus replication by removing K48-linked ubiquitination of HBV core/precore and upregulates HNF4ɑ expressions by inhibiting the ERK1/2/mitogen-activated protein kinase pathway
Source: Cell Mol Life Sci. 2023 Oct 28;80(11):336. doi: 10.1007/s00018-023-04995-2 (PMC10613150; doi:10.1007/s00018-023-04995-2)

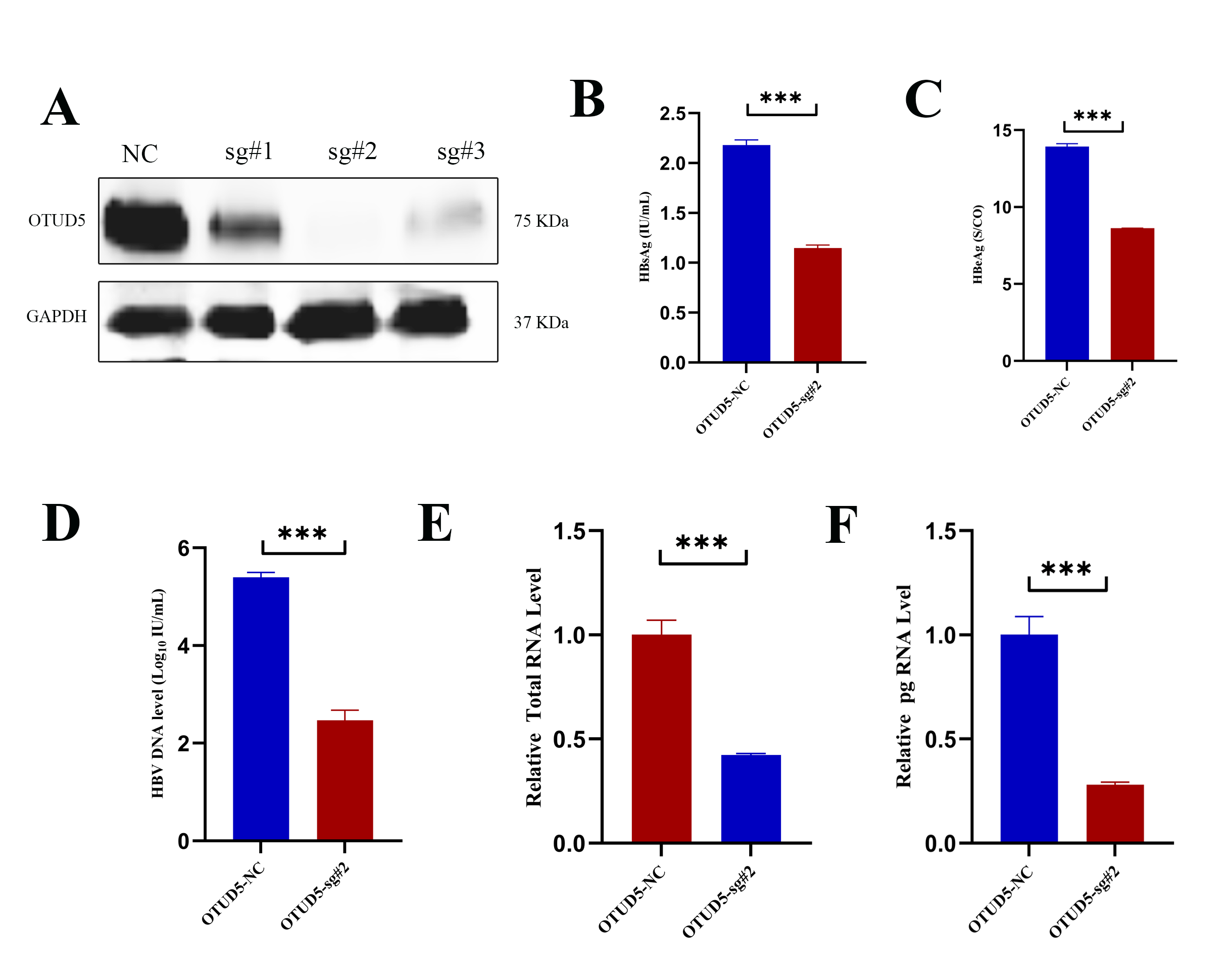

Supplement: Supplementary file 1 — Knockout of OTUD5 inhibited HBV transcription and replication in HepG2. 2.15 cell lines. HepG2.2.15 cells were infected with OTUD5-sg plasmid using CRISPR–cas9 technology and mock vector as the negative control, and Western blot results showed the knockout effect of OTUD5 (A). The concentration of HBsAg and HBeAg was decreased in OTUD5-sg#2 compared with a negative control in supernatant (B–C, F). The expression of pg RNA and HBV total RNA significantly decreased in HepG2.2.15 OTUD5-sg#2 cells compared with negative control (D-E) (TIF 360 KB) [file 18_2023_4995_MOESM1_ESM.tif]

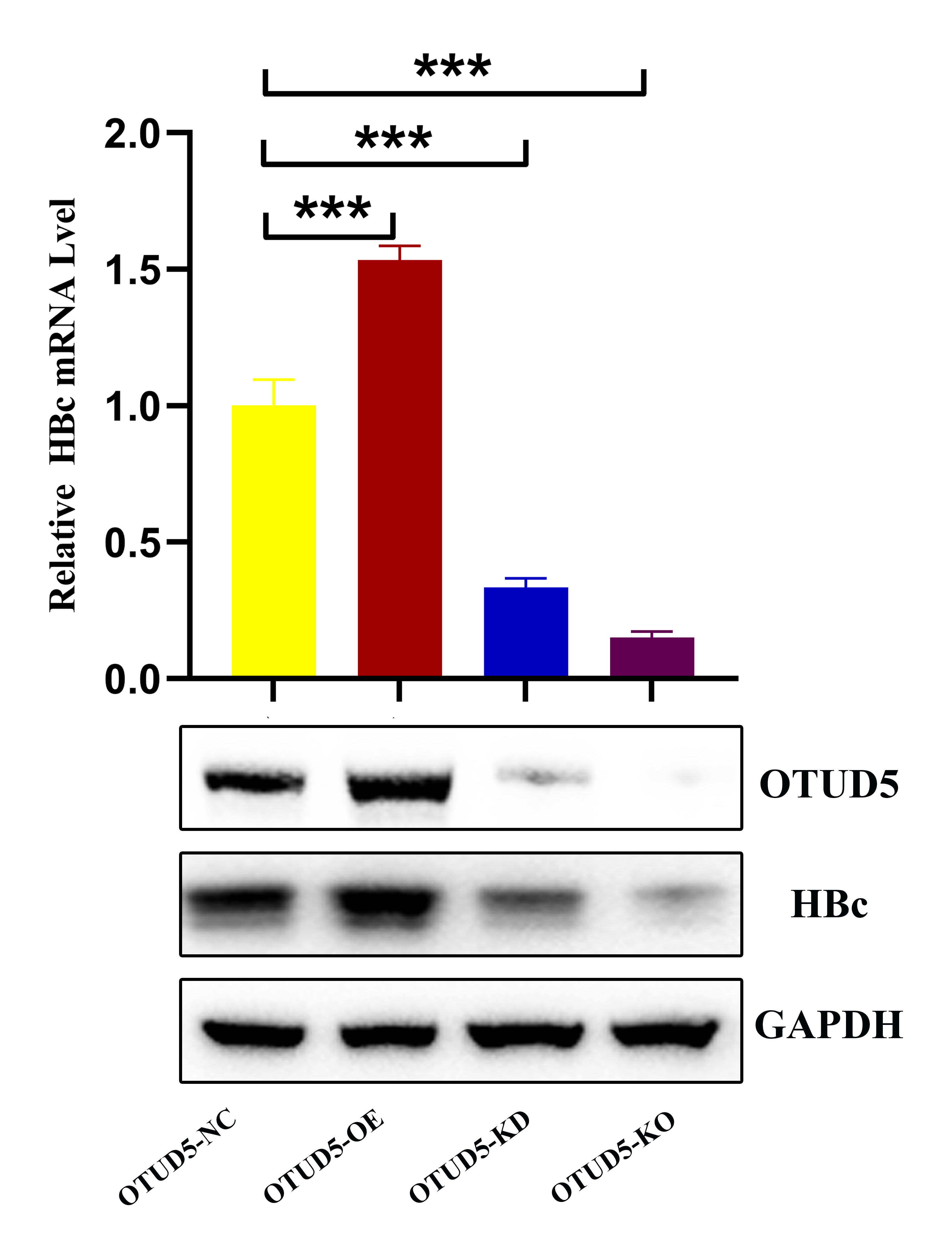

Supplement: Supplementary file 2 — Endogenous HBV core at protein and mRNA level in the same experiment, in control, OTUD5 OE, KD and KO. HepG2.2.15 cells were infected with OTUD5-control, OTUD5-overexpression, OTUD5-sh and OTUD5-sg plasmids. Western blot results showed the protein levels of OTUD5 and HBc, PCR result showed the relative expression of HBc mRNA (TIF 938 KB) [file 18_2023_4995_MOESM2_ESM.tif]

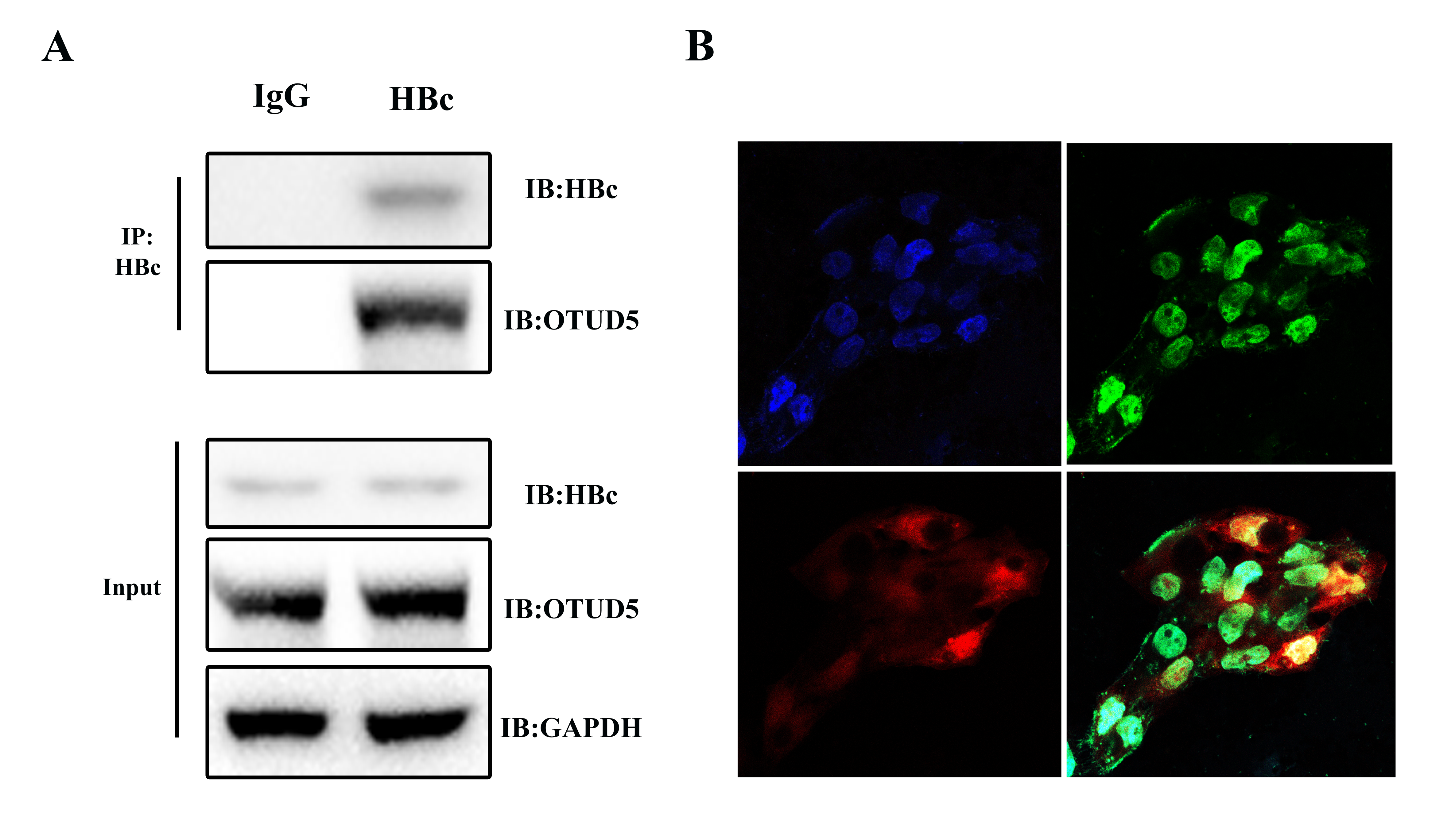

Supplement: Supplementary file 3 — Interaction experiments of endogenous HBV core and OTUD5. Endogenous HBV core and OTUD5 interaction experiments were performed in coimmunoprecipitation and colocalization assays. HepG2.2.15 cells were harvested after treatment with MG132 for 8 h. Cellular lysates were subjected to IP with anti-HBc and IB with anti-OTUD5 (A). Confocal microscopy analysis of colocalization of endogenous OTUD5 with HBc. HepG2.2.15 cells were incubated with anti-HBc and anti-OTUD5 overnight, then incubated with a secondary antibody conjugated to Alexa Fluor 637 or Alexa Fluor 488 for fluorescent staining (B) (TIF 6957 KB) [file 18_2023_4995_MOESM3_ESM.tif]

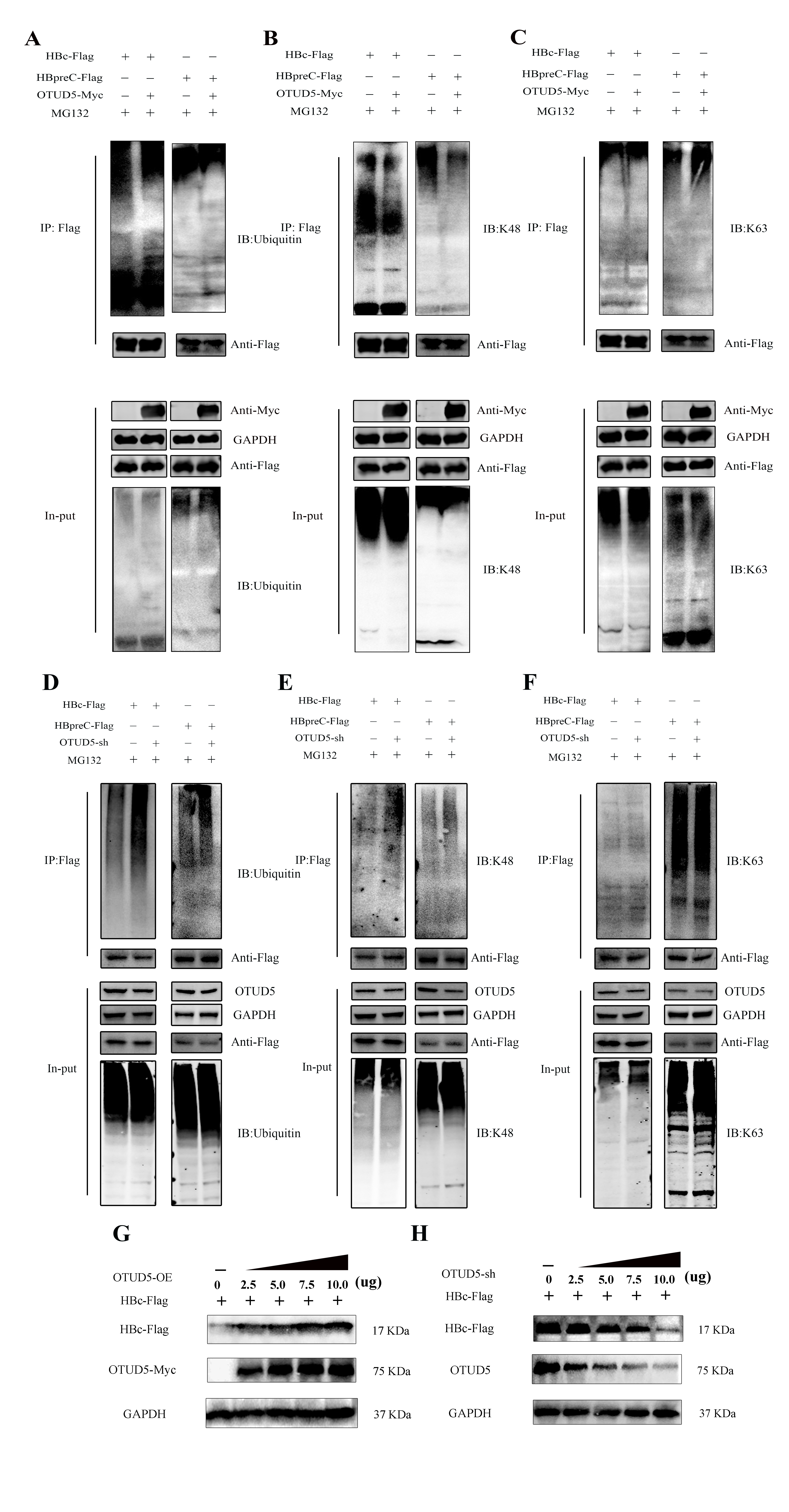

Supplement: Supplementary file 4 — OTUD5 inhibited the hepatitis B precore/core proteins degradation through the proteasome pathway in a ubiquitin-independent manner. Hepatitis B precore/core plasmids with flag tag and OTUD5 overexpression plasmid with myc tag were co-transfected into HEK293T cells. After transfection, the cells were treated with MG132 for 8 h, lysates immunoprecipitation and whole cell lysates analysis of the deubiquitination of HBc were performed by Western blot (A-C). Hepatitis B precore/core plasmids with flag tag and OTUD5 knockdown plasmid were co-transfected into HEK293T cells. After transfection, the cells were treated with MG132 for 8 h, lysates immunoprecipitation and whole cell lysates analysis of the deubiquitination of HBc were performed by Western blot (D-F). HEK293T cells were co-transfected with HBc-Flag and different doses of OTUD5 overexpression or OTUD5 knockdown plasmids. After 72 h, the cells were lysed and HBc protein levels were analyzed by Western blot using the anti-flag antibody (G–H) (TIF 5399 KB) [file 18_2023_4995_MOESM4_ESM.tif]
